# 

**Published:** 1891-03-21

**Authors:** 


### NOTICE TO CORRESPONDENTS.

All MS., Letters, Books for Review, and other matters intended for the Editor should be addressed The Editor, The Lodge, Porchester Square, London, W.

The Editor cannot undertake to return rejected M.S., even when accompanied by stamped directed envelope.

**Notice to Advertisers and Subscribers.**

All Advertisements, Orders for Copies of The Hospital, and Business Communications should be addressed to The Publisher, *not to the Editor*, The Hospital, 140, Strand, W.C.

The Cost of Subscription, post free, is as follows:—Per week, 1½d.; per quarter, 1s. 8d.; per half-year, 3s. 3d.; per annum, 6s. 6d. Foreign:—Per annum, 8s. 8d.; payable, in all cases, in advance.

### Scraps and Gleanings.

The cost of the new Sick Hospital for Dundee is estimated at £22,000.

Mr. Joseph Ruston has offered to defray the cost of a much-needed children's ward at the Lincoln County Hospital.

Permission has been given for the formation of Volunteer Medical Staff Corps in Glasgow, consisting of two bearer companies.

The Duke of Connaught has promised to preside at a dinner on behalf of the funds of the North-Eastern Hospital for Children, Hackney Road, on Friday, May 15th.

An epidemic of infantine cholera is stated to have broken out in several villages near Pressburg, Hungary, 70 per cent. of the cases ending fatally within twenty-four hours.

The annual report for 1890 of the Llandrindod Wells Cottage Hospital shows that 153 patients were admitted, this being the largest number yet recorded.

The second annual report of the Cottage Hospital, Blandford, is satisfactory in every way. Fifty-five patients were admitted during the year. The financial statement shows a balance in the hands of the Treasurer of £167 3s. 10d.

A number of applicants for registration by the Worshipful Company of Plumbers were examined at the Guilds Institute, on the 28th ult. The examinations while not unduly severe were such as to thoroughly test the manual skill and theoretical knowledge of the candidates, of whom only one-fifth succeeded in passing the full examinations.

During the year 1890 the number of in-patients treated in the Aberdeen Royal Infirmary was 2,210, being a slight decrease on the numbers for the two previous years. There is an adverse balance of £230 3s. 4d. against the institution, due to the cost of laundry work which has to be done at a distance, and to a material increase under the head of medical outlay. The amount subscribed towards the Jubilee Extension Scheme has reached £30,598 16s. 4d. The amount already expended is £17,099 10s. 3d.

The Duke and Duchess of Westminster have promised to open the annual sale of inmates' work at the Royal Hospital for Incurables, West Hill, Putney Heath, on Tuesday, June 1[illegible]th next.

The Town Association of Torquay have decided to have in the town a "Battle of Flowers" on Thursday in Easter week. The flowers will be distributed among the hospitals of the town and district, and the proceeds will be given to the Torbay Hospital.

During the past year much has been done to extend the usefulness of the Belfast Lying-in Hospital. A trained lady has been appointed as Matron and nurse combined, and the hospital has been supplied with many improved medical appliances, and is now in a thoroughly satisfactory working condition. The nursing of the poor in their own homes has been resumed, a trained lady nurse having been appointed to this post last.

A special Indian Committee has been formed in connection with the International Congress of Hygiene to be held in London in August.

A despatch from St. Louis states that yellow fever is raging in the Cameroons, and that grave fears are entertained of an outbreak at St. Louis.

The camphor tree is now being cultivated on an extensive scale in Florida. It grows rapidly there, and seems to flourish on any kind of soil.

The Mayor of Florence has officially informed the Municipal Council that the public health is now restored to its normal condition, owing to the sanitary measures which have been adopted. The special commission appointed to examine the water supply has pronounced that the water now used for drinking purposes in the city is of pure and excellent quality.

The proceeds of the three dances in aid of the funds of the North-west London Hospital amounted to £396 16s., and, in addition to this sum, £68 1s. in special donations has been handed over to the Committee of Management.

The annual meeting of the London Temperance Hospital supporters took place at Hampstead last week. During the past year the out-patients numbered 3,282, and the in-patients 753. The receipts amounted to £4,415, and the expenditure to £4,376.

A fire took place on the 8th inst. at St. Anne's Home, the Bowden branch of the Manchester Hospital for Consumption. The flames broke out in the old portion of the building, where there were 17 beds; only two of these were occupied at the time, however, the other patients being in the day-room. Eventually the fire was subdued by the local brigade, and the building was saved. Much damage was done to the lower rooms.

Mr. F. Webb has been unanimously elected to the post of House Surgeon at the Doncaster Infirmary in the place of Mr. T. Lund, who has resigned.

### BOOKS RECEIVED.

Simpkin, Marshall, and Co.

"Evolution: The Work of a Great Intelligence." W. A. Part I.

Trow's Printing and Bookbinding Co. (New York).

"The Rotary Element in Lateral Curvature of the Spine." A. B. Judson, M.D. (Reprinted for the *Medical Record*.)

H. K. Lewis.

"The Thermometer in Obstetrics and Gynæcology." A. D. Leith Napier, M.D. (Price 1s. 6d.)

"On the Use of the Oil of the Eucalyptus Globulus, combined with other Antiseptics in the Treatment of Scarlet Fever and all Infectious Diseases." J. Brendon Curgenven, M.R.C.S. (Price 1s.)

"Notes on the Examination of the Sputum, Vomit, Fæces, and Urine." Sidney Coupland, M.D., F.R.C.P. Price 1s.

Livingstone, E. and S. (Edinburgh).

"Materia Medica," Part II.; and "Anatomy: Head and Neck." ("Catechism" Series.) Price 1s. each.

### THE STUDENT OF MEDICINE.

[All communications for this Supplement should be addressed to THE EDITOR OF THE HOSPITAL, at 140, Strand, with the words, "Students' Column," written in the left hand top corner of the envelope.]

### NOTES FROM THE SCHOOLS.

### Edinburgh University.

The Edinburgh University Union Dramatic Society held its first festival on January 23rd and 24th, in the Albert Hall. There have been dramatic festivals before, but this was the most interesting, being the first of the new Society's performances, and because, with the exception of the actresses and Mr. Shiels' substitute, it was entirely done by students. The principal piece, Leicester Vernon's military comedy-drama, "The Lancers," was an unqualified success. Mr. W. Lyall Wilson made a very good Colonel de Franc-Epeé, and Mr. J. Wilson Shiels put much feeling into the rendering of Victor de Courcy. Unfortunately, he was unable to play on the 24th, but Mr. R. C. Morrison made an excellent substitute. Mr. J. G. Walker, as Troop-sergeant-major Moustache, acted uncommonly well. Blanquet (Mr. D. Lander Lindsay) was at times slightly too boisterous, but, on the whole, was very good. The part of Eugene gave Mr. A. Stodart Walker no scope: his song, "Soldier Jim," was remarkable for its clear articulation. Of the actresses, Miss Alice Brickmann, as Estelle Duvernay, acted and looked uncommonly well; so did Miss Mary Bernard, as Madame Pomponne. Miss Marie Watson was very effective as "my brother the kurnel's" cast-iron sister. The second piece, an extravaganza, "Buz Buz," was poor in itself. Mr. J. G. Walker was undoubtedly best as court physician; and Mr. D. Lander Lindsay, as court jester, was also good. Mr. W. Campbell Lahore was acting manager. The University orchestra performed during the evening.

The Union Committee have consented to allow smoking in the Debating Hall of the Union for a month as an experiment, an arrangement which already seems to be appreciated.

Mr. Lawson Tait, on February 3rd, lectured on the surgical treatment of gall stones to Professor Grainger Stewart's Practice of Medicine Class.

The managers of the Royal Infirmary have decided to admit lady medical students to the wards of that institution. "Mixed" cliniques, except in certain cases, are not to be allowed, nor are the present cliniques to be interfered with.

A smoking concert was held in the Union on February 7th, arranged by the Students' Representative Council. Mr. Goschen, the Lord Rector, was asked to take the chair, but was unable to leave London. Lord Stormouth Darling took the chair in his stead, and made a very good substitute with his story-telling. Professors Blackie, Corsar, Ewart, Masson, and Rankine, Mr. Baird, M.P., and Dr. J. Greig were also present. The piece of the evening was Herr Gallrein's 'cello solo, which received a well-deserved encore. Mr. Ehrke received an encore for his violin solo. The "Dodos," with their banjos, and Mr. Clapperton, on the French horn, were well received. Mr. Stodart Walker and Mr. Guthrie deserved their encores. Mr. Lyall Wilson's recitation, "Kissing Cup," was splendidly given, and received an encore.

### Dental Hospital, Liverpool.

The annual general meeting of the above Society was held on Wednesday, January 21st, a large gathering of members and friends being present. The election of officers for the current year was as follows: Mr. T. Mansell, L.D.S., President; Mr. R. H. Bates, L.D.S., Vice-President; Mr. W. H. Silmour, Treasurer; Mr. F. C. Dopson, Secretary. Council, Mr. L. J. Osborn, Mr. F. R. Suyler, Mr. J. H. Burroughs. The Secretary and Treasurer read the first annual report, which were proved to be very satisfactory, showing great progress of the Society. Mr. D. W. Parsons then read a paper, entitled "Mechanical Dentistry and the Last London Examination," after which a very interesting discussion took place.

### Middlesex Hospital Medical Society.

At a meeting, held on Thursday evening, January 29th, Mr. T. Carwardine read a paper entitled "Phrenology and the Localisation of Function." He introduced the subject by reference to ancient ideas and the remains of them in modern speech, and to the psychical resemblances and differences existing between man and animals. An outline of phrenology was given, comprising a short history and a description of some of the bumps. The assumptions that the convolutions represent "organs," and that the true form of the brain can be understood from that of the skull, were next criticised in the light of anatomy. The more reliable results of physiological experiments were then considered and compared with phrenology, and it was

THE STUDENT OF MEDICINE—(*continued*).

pointed out that some mental functions are beyond the range of physics and physiology. The origin of the will was, in conclusion, discussed, and views expressed as to the relations between the mind of man and evolution, environment and energy. The President and Mr. Holding took part in the subsequent discussion. Mr. F. A. Wagstaffe then read the notes of a case of "Ulcerative Endocarditis," illustrative of how sometimes only slight *post-mortem* lesions are found after well-marked symptoms of disease during life.

### APPOINTMENTS.

At Guy's Hospital, Herbert Vigers Hickman, L.R.C.P. Lond., M.R.C.S. Eng., has been appointed ophthalmic assistant. At the Wirral Children's Hospital, Birkenhead, James D. C. Allan, M.B., C.M. Edin., has been appointed house surgeon. At the Royal Victoria Hospital, Bournemouth, Theodore Lund, M.B., B.S. Durh., has been appointed surgeon and secretary. At the Doncaster Infirmary, Frank Webb, L.R.C.P. Lond., M.R.C.S. Eng., has been appointed house surgeon. At the London Temperance Hospital, Hampstead Road, S. L. Hinde, L.S.A., has been appointed house surgeon. At the Chichester Infirmary, Stanley de Butts, M.R.C.S. Eng., L.R.C.P. Lond., has been appointed house surgeon. At the North Staffordshire Infirmary and Eye Hospital, Jermyn F. Gillett, B.A., M.B., B.C. Cantab., has been appointed assistant house surgeon. At the Scarborough Hospital and Dispensary, Victor Hodgson, L.R.C.P. Lond., M.R.C.S. Eng., has been appointed house surgeon. At the Bear Yard Workhouse, Strand Union, J. R. Harris, M.D. Brussels, L.R.C.P. Edin., M.R.C.S., has been appointed medical officer. At the Great Northern Hospital, Holloway Road, Raymond Johnson, M.B., B.S. Lond., F.R.C.S. Eng., has been appointed surgeon to the out-patients; and W. A. Malcolm, M.B., C.M. Edin., casualty officer. At the Hospital for Women, Chapel Ash, Wolverhampton, John Allan Lycett, M.D. St. And., L.R.C.P. Lond., M.R.C.S. Eng., has been appointed acting surgeon. At the Bristol General Hospital, Charles A. Morton, F.R.C.S. Eng., has been appointed Registrar. At the Royal Infirmary, Manchester, E. S. Reynolds, M.D. Lond., M.R.C.P., M.R.C.S., has been appointed superintendent medical officer. At the Royal Hospital for Diseases of the Chest, City Road, Fred. A. Smith, B.A. Oxon., M.R.C.P. Lond., M.R.C.S., has been appointed assistant physician. The Royal Mail steamship "Spartan," T. A. Ollivant Langston, L.R.C.P. Lond., M.R.C.S. Eng., has been appointed surgeon. Mr. E. Leonard Lees, M.B., C.M., M.R.C S., has been appointed medical registrar of the Bristol Hospital for Sick Children and Women.

### VACANCIES.

The following vacancies are announced this week, the amount of salary in each case being given after the name of the institution:

**Resident Medical Officers.**

Belgrave Hospital for Children, 79, Gloucester Street, S.W., House Surgeon—Board, lodging &c.
Bethlem Hospital, S.E., Two Resident Clinical Assistants.
Bradford Infirmary and Dispensary, Dispensary Surgeon—Salary £100, &c.
Chelsea Hospital for Women, Fulham Road, S.W., Medical Officer—Salary £80, &c. Also Clinical Assistant—Fee of £3 3s. for three months.
County Asylum, Rainhill, near Liverpool, Assistant Medical Officer—Salary £100, &c.
District Infirmary, Ashton-under-Lyne, House Surgeon—Salary £90, &c.
Jaffray Suburban Branch of the General Hospital, Gravelly Hill, near Birmingham, Resident Medical Officer—Salary £150, &c.
King's Lynn Hospital, House Surgeon and Secretary—Salary £80, &c.
Liverpool Northern Hospital, Assistant House Surgeon—Salary £70, &c.
Liverpool Dispensaries, Assistant Surgeon—Salary £80, &c.
Metropolitan Asylums Board, Clinical Assistant for South-Eastern Fever Hospital, New Cross Road, S.E.
Queen Charlotte's Lying-in Hospital, Marylebone, N.W., Resident Medical Officer for four months—Salary £60.
Royal Bath Hospital and Rawson Convalescent Home, Harrogate, House Surgeon and Secretary—Salary £80, &c.
St. Thomas's Hospital, S.E., Resident Assistant Physician.
Teignmouth, Dawlish, and Newton Dispensary and Convalescent Home, House Surgeon—Salary £40, &c.
Township of Manchester, Assistant Medical Officer for the Workhouse at Crumpsall—Salary £100, &c.
West London Hospital, Hammersmith Road, W., House Physician and House Surgeon.

**Non-Resident Medical Officers.**

Dental Hospital of London, Four Demonstrators—Honorarium £50.
Guy's Hospital Medical School, Two Anæsthetists for the Dental department.
King's College, London, Demonstrator of Physiology.
Loddon and Clavering Union, Loddon, near Norwich, Medical Officer and Public Vaccinator—Salary £55, and fees.

### The Hospital Advertiser.

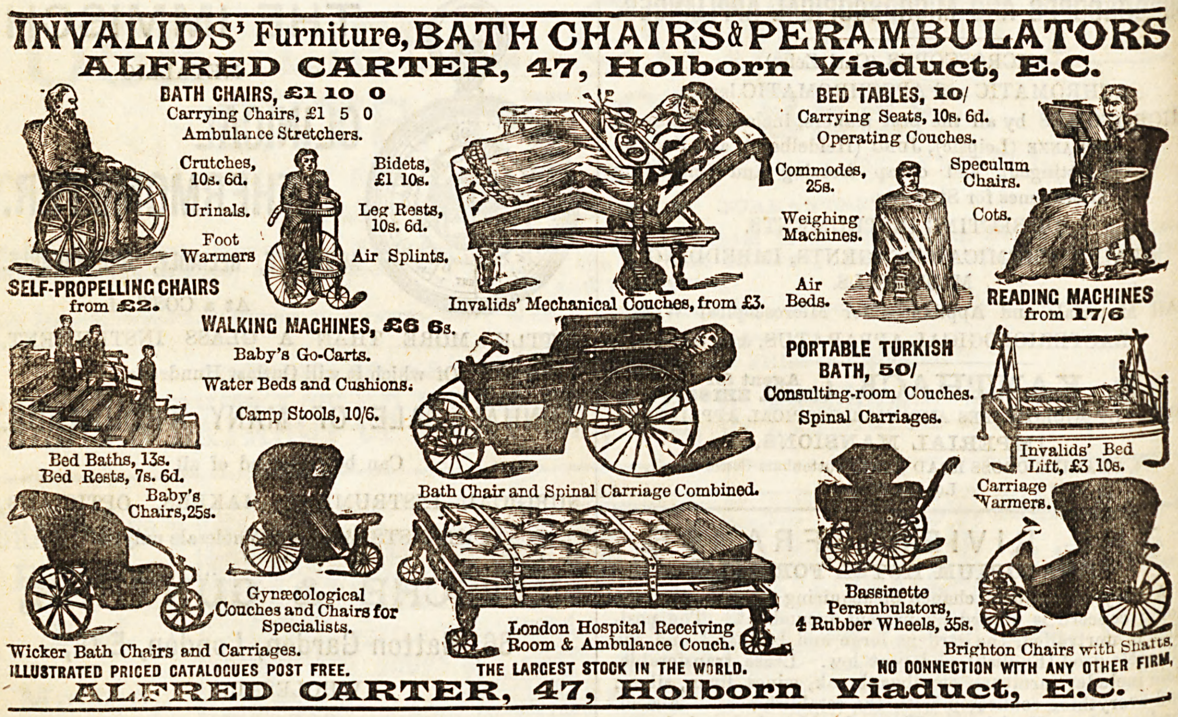

ALL GOOD CHEMISTS SUPPLY.
PETER MÖLLER'S Being Absolutely Pure, and in Capsuled Bottles, it is consequently free from the large quantity of WATER necessarily in emulsions usually charged for at the highest price of oil. COD LIVER OIL.

### THE STUDENT OF MEDICINE—(*continued*).

Newcastle-on-Tyne Dispensary, Visiting Medical Officer.—Salary £120.

Norfolk and Norwich Hospital, Norwich, Physician.

St. George's and St. James's Dispensary, King Street, Regent Street, W., Surgeon.

St. Pancras and Northern Dispensary, 126, Euston Road, Honorary Physician.

Township of Manchester, Visiting Surgeon for the Workhouse Infirmary at Crumpsal—Salary £160.

West London Hospital, Hammersmith Road, W., Assistant Surgeon.

### PASS LISTS.

### Army Medical Staff.

THE following list has been published of successful candidates at the recent competitive examination for commissions in the Medical Staff of Her Majesty's Army:

| | Marks. | | Marks. |
|---|---|---|---|
| Porter, F. J. W. ... ... | 3135 | Hardy, W. E. ... ... ... | 2780 |
| Robinson, O. L. ... ... | 3025 | Begbie, F. W. ... ... ... | 2770 |
| Brown, F. J. ... ... ... | 2960 | Burtchaell. C. H. ... ... | 2760 |
| Healy, C. J. ... ... ... | 2960 | Lenehan, T. J ... ... ... | 2760 |
| Stalkart, C. E. G. ... ... | 2930 | McDowell, F. ... ... ... | 2760 |
| Gibbard, T W.... ... ... | 2925 | Faichnie, N. ... ... ... | 2755 |
| Buist, H. J. M. ... ... | 2905 | MacCarthy, J. A. O. ... ... | 2695 |
| Duggan, C. W.... ... ... | 2880 | Gerrard, J. J. ... ... ... | 2690 |
| Brogden, J. E.... ... ... | 2815 | Stanistreet, G. B. ... ... | 2680 |
| Austin, J. H. E. ... ... | 2780 | Jameson, J. C.... ... ... | 2670 |

### Indian Medical Service.

There were 59 candidates for 21 appointments, and 57 were reported qualified.

| | Marks. | | Marks. |
|---|---|---|---|
| Burton-Brown, F. H.... ... | 3625 | Frost, G. H. ... ... ... | 3035 |
| Lumsden, J. S. S. ... ... | 3340 | Smith, S. B. ... ... ... | 3030 |
| Deare, B. H. ... ... ... | 3330 | Graves, D. H. McD. ... ... | 3020 |
| Brown, A. T. ... ... ... | 3215 | Penny, J. ... ... ... | 3020 |
| Bird, R., M.D.... ... ... | 3180 | Ewens, G. F. W., M.D. ... | 3010 |
| Oldham, B. C. ... ... ... | 3165 | Henvey, W. ... ... ... | 3010 |
| Wilkinson, E. ... ... ... | 3160 | Pridmore, W. G. ... ... | 3010 |
| Duer, C. ... ... ... ... | 3155 | Irvine, J. W. ... ... ... | 3005 |
| Wood, H. S. ... ... ... | 3125 | Dallas, S. A. C. ... ... | 2995 |
| Entrican, J., M.D. ... ... | 3090 | Palk, C. H. L. ... ... ... | 2975 |
| Donovan, C., M.D. ... ... | 3050 | | |

### ATHLETICS.

### Football.

ASSOCIATION RULES.

LONDON SENIOR CUP (Final Tie).—*Royal Arsenal v. St. Bartholomew's Hospital*.—This match was played on Saturday, March 7th, at the Oval, Kennington, in the presence of over 5,000 visitors. It was expected that a well-contested game would ensue, and many believed that Bart.'s men would win; this, however, was unfortunately not the case. The spectators were well supplied with musical (?) instruments of various kinds, and throughout the ninety minutes' play most discordant sounds were heard. The Medicals were the first to take the field, and had a few minutes practice before their opponents arrived on the scene. At twenty-five minutes past three the ball was kicked off by Dixon, and a spell of give-and-take play followed. From the goal kick the Medicals attacked on the right, but Fry, getting the ball, finished up by a shot at goal. Coulby was, however, equal to the occasion, and Fernie clearing the lines, Hogarth went away. The last was next conspicuous, and after a grand dribble crossed to Bond. At half-time the game stood as follows: Royal Arsenal, five goals; St. Bart.'s, none. After crossing over, the Medicals, who had the advantage of the wind, took the ball into their opponents' quarters, and their unsuccessful attempt to score, Wilson sending the ball behind. Bart.'s men were attacked, and Coulby was called upon to save a hard shot. Christmas then forced a corner off Mackintosh; the ball was well placed, but Stewart headed over. Fernie got the ball away, and Hogarth, with a good run nearly the length of the field, if he had been supported should have scored. The Arsenals again attacked, and a free kick falling to them enabled Fry to register a further goal. Fast play ensued, but nothing more was scored, the Medicals being defeated by six goals to none.

MARCH 12TH.

HOSPITALS' ASSOCIATION CUP.—*St. Thomas's* won their tie against *King's* by two goals to none, at Leyton.

MARCH 13TH.

The tie in the penultimate round of the competition for the Hospitals' Association Cup between *St. Bartholomew's* and *Guy's* was played at Leyton. St Bartholomew's were successful by four goals to two. The final tie rests between St. Bartholomew's (holders) and St. Thomas's.

MARCH 14TH.

*Cambridge Past and Present* beat *Oxford Past and Present*, at the Oval (seven goals to two). *Guy's Hospital* were beaten by *Chiswick Park*, at Chiswick (five goals to nil). *St. Bartholomew's* drew with *Old Selways*, at Leyton (one goal to one).

RUGBY, MARCH 14TH.

*St. Thomas's Hospital* v. *R.I.E.C.*—This match was played at Cooper's Hill, and resulted in a victory for the Hospital by a goal to nil. The game was keenly contested throughout, and twenty minutes from the start, after a splendid run, Toller passed Prain, who got in. Although there was some splendid play, nothing further was scored. *University College Hospital* was beaten by *Hampstead*, at Neasden (one goal three tries to nil). *Middlesex Hospital* drew with *Lennox*, at Dulwich (nil to nil).

